# Encapsulation of the dual FLAP/mPEGS-1 inhibitor BRP-187 into acetalated dextran and PLGA nanoparticles improves its cellular bioactivity

**DOI:** 10.1186/s12951-020-00620-7

**Published:** 2020-05-14

**Authors:** Blerina Shkodra, Christian Kretzer, Paul M. Jordan, Paul Klemm, Andreas Koeberle, David Pretzel, Erden Banoglu, Stefan Lorkowski, Maria Wallert, Stephanie Höppener, Steffi Stumpf, Antje Vollrath, Stephanie Schubert, Oliver Werz, Ulrich S. Schubert

**Affiliations:** 1 Laboratory of Organic and Macromolecular Chemistry (IOMC), Friedrich Schiller University Jena, Humboldtstraße 10, 07743 Jena, Germany; 2 Jena Center for Soft Matter (JCSM), Friedrich Schiller University Jena, Philosophenweg 7, 07743 Jena, Germany; 3 Department of Pharmaceutical/Medicinal Chemistry, Institute of Pharmacy, Friedrich Schiller University Jena, Philosophenweg 14, 07743 Jena, Germany; 4 Department of Pharmaceutical Chemistry, Faculty of Pharmacy, Gazi University, Etiler, Yenimahalle, 06330 Ankara, Turkey; 5 Institute of Nutritional Sciences, Friedrich Schiller University Jena, Dornburger Straße 25, 07743 Jena, Germany; 6 Department of Pharmaceutical Technology and Biopharmacy, Institute of Pharmacy, Friedrich Schiller University Jena, Lessingstraße 8, 07743 Jena, Germany; 7 Michael Popp Reseach Institute, University of Innsbruck, Mitterweg 24, 6020 Innsbruck, Austria

**Keywords:** Acetalated dextran, PLGA, Nanoparticles, Leukotriene biosynthesis, FLAP inhibitor, MPGES-1, Dual inhibitor, BRP-187

## Abstract

**Background:**

Dual inhibitors of the 5-lipoxygenase-activating protein (FLAP) and the microsomal prostaglandin E_2_ synthase-1 (mPGES-1) may exert better anti-inflammatory efficacy and lower risks of adverse effects versus non-steroidal anti-inflammatory drugs. Despite these advantages, many dual FLAP/mPGES-1 inhibitors are acidic lipophilic molecules with low solubility and strong tendency for plasma protein binding that limit their bioavailability and bioactivity. Here, we present the encapsulation of the dual FLAP/mPGES-1 inhibitor BRP-187 into the biocompatible polymers acetalated dextran (Acdex) and poly(lactic-*co*-glycolic acid) (PLGA) via nanoprecipitation.

**Results:**

The nanoparticles containing BRP-187 were prepared by the nanoprecipitation method and analyzed by dynamic light scattering regarding their hydrodynamic diameter, by scanning electron microscopy for morphology properties, and by UV–VIS spectroscopy for determination of the encapsulation efficiency of the drug. Moreover, we designed fluorescent BRP-187 particles, which showed high cellular uptake by leukocytes, as analyzed by flow cytometry. Finally, BRP-187 nanoparticles were tested in human polymorphonuclear leukocytes and macrophages to determine drug uptake, cytotoxicity, and efficiency to inhibit FLAP and mPGES-1.

**Conclusion:**

Our results demonstrate that encapsulation of BRP-187 into Acdex and PLGA is feasible, and both PLGA- and Acdex-based particles loaded with BRP-187 are more efficient in suppressing 5-lipoxygenase product formation and prostaglandin E_2_ biosynthesis in intact cells as compared to the free compound, particularly after prolonged preincubation periods.

**Graphical Abstract:**

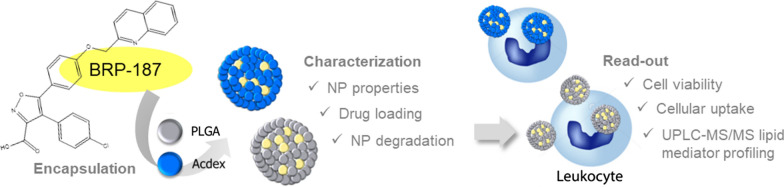

## Background

Inflammation is a physiological reaction of the body to fight harmful invaders and to restore damaged tissue. However, if inflammation persists and the body cannot return to homeostasis, chronic inflammatory diseases such as arthritis, Alzheimer´s disease or arteriosclerosis can evolve [1]. Inflammation is initialized and maintained by prostaglandins (PG) and leukotrienes (LT) that are biosynthesized from arachidonic acid (AA) [2]. For PG formation, AA is first converted by cyclooxygenase-1 and -2 (COX-1/2) to the intermediate prostaglandin H_2_ (PGH_2_), which is subsequently metabolized by specific PG synthases into different bioactive prostanoids. Among them, prostaglandin E_2_ (PGE_2_) is massively produced by microsomal prostaglandin E_2_ synthase-1 (mPGES-1) and is most relevant for inflammation, while other prostanoids (e.g. prostaglandin I_2_, thromboxane A_2_) are of importance for homeostatic processes, e.g., regulation of blood pressure and platelet aggregation [3]. Current anti-inflammatory therapies include non-steroidal anti-inflammatory drugs (NSAIDs) that prevent inflammation by blocking PG biosynthesis via inhibition of COX-1/2. However, inhibition of COX enzymes is associated with side effects since the formation of all prostanoids is blocked and AA is preferably metabolized to pro-inflammatory LT [4]. The formation of LT from AA is initialized by 5-lipoxygenase (5-LO) together with 5-LO-activating protein (FLAP), with the latter facilitating access of 5-LO to AA [5]; both proteins are pursued as molecular targets in the development of anti-inflammatory drugs [6].

BRP-187 (4-(4-chlorophenyl)-5-[4-(quinoline-2-ylmethoxy)phenyl] isoxazol-3-carboxylic acid) is a dual inhibitor of mPGES-1 and FLAP suppressing the formation of pro-inflammatory PGE_2_ and LTs [7]. This pharmacological approach is proposed to be more efficacious and associated with fewer adverse effects than inhibition of the COX pathway [8]. However, BRP-187 is an acidic lipophilic molecule with low water solubility and a strong tendency for plasma protein binding, implying the need for new technological approaches to overcome these disadvantages. Encapsulation of small molecule drugs that exhibit a low solubility into biodegradable polymers (polyesters or polyketals) can improve their bioavailability [9, 10]. Here, we attempted to encapsulate BRP-187 into polymer-based nanoparticles (NPs) using poly(lactic-*co*-glycolic acid) (PLGA) and acetalated dextran (Acdex). These polymers were selected because they are biocompatible materials able to successfully encapsulate hydrophobic drugs, and as such, they could facilitate an increase in the retention time of the drug in the plasma [11]. As a polyester, PLGA is enzymatically hydrolyzed into physiological metabolites—lactate and glycolate [12], hence, it is used and widely investigated as a biomaterial for drug delivery [13–15]. The properties of PLGA are influenced by the ratio of lactic to glycolic acid units, with a 50:50 composition showing the fastest degradation [16]. An alternative to PLGA is Acdex, a recently developed dextran derivative offering favorable properties as carrier for drugs with low solubility [17]. Acdex is composed of acyclic and cyclic acetal groups, with the acyclic acetals degrading faster than the cyclic acetal groups [17]. As a consequence, the degradation behavior of Acdex can be fine-tuned by varying the degree of substitution of the cyclic vs. acyclic acetal groups on the dextran backbone [18]. Most importantly, under slightly acidic conditions (e.g. pH 5.5), the acetal groups are cleaved, resulting in biocompatible, water-soluble dextran while instantly releasing the cargo [19]. In brief, the main advantage of Acdex lies in its facile synthesis (i), in the possibility to design formulations with a desired release profile by varying the cyclic vs. acyclic acetal groups in the dextran backbone (ii), and in its sensitivity to low pH levels—typical conditions of inflamed tissues and endosomal compartments [20]. In this view, both polymer formulations are intended for parenteral administration, with Acdex being suitable as an instant-release formulation, whereas PLGA might be suitable for extended-release formulations. In addition, based on the longer degradation time of the PLGA NPs in tissue (> 40 days), depot formulations for local administration routes (e.g. intra-muscular or intra-articular for rheumatoid arthritis) could also be considered.

In this study, formulations of BRP-187-containing NPs of 130 to 230 nm using Acdex and PLGA as biodegradable encapsulating materials were prepared. The properties of these NPs were analyzed including the degradation behavior, and the FLAP/mPGES-1-inhibitory efficiency of BRP-187-containing NPs was evaluated in comparison to the free compound in different human primary leukocytes.

## Results and discussion

The formulation parameters were designed to produce stable monodisperse particles of 100 to 200 nm and with high drug loading. The excipients used in the formulation were selected after careful consideration based on previous data from our lab, literature, and most importantly based on the technical requirements of the International Council for Harmonization (ICH) guidelines for pharmaceuticals for human use. Acetone was used as organic phase for the following reasons: It is a good solvent for both polymers (i); it is miscible with water, which is a pre-requisite for solvents used in nanoprecipitation (ii) [21]; it can be easily removed from the formulation by evaporation at room temperature (iii); and according to the ICH, acetone is a Class 3 solvent with a low toxicity [22]. Next, for the solubilization of the drug, due to the high lipophilicity of BRP-187, the choice of the solvent was limited only to dimethylsulfoxide (DMSO) and dimethylformamide (DMF). Thus, considering that DMF is more toxic than DMSO (residual concentration limit 880 ppm vs. 5000 ppm, respectively), the latter was selected for the formulation [22]. Meanwhile, the residual amount of DMSO in our NP formulations was < 250 ppm. Furthermore, the volumetric ratio of organic-to-aqueous phase was kept at 1:8 to produce particles < 200 nm with an encapsulation efficiency EE > 50%, higher volumetric ratios produce NPs > 200 nm [23]. Partially-hydrolyzed poly(vinyl alcohol) (PVA) was used as surfactant and cryoprotectant to prevent aggregation during purification and lyophilization, respectively. We have previously demonstrated that PVA provided a superior stability of PLGA NPs than poloxamers and polysorbates at concentrations < 0.5%, and no toxicity was evident even at 100-fold higher concentrations [10].

### Characterization of NPs

Initial experiments revealed that NPs prepared by the nanoprecipitation method had a higher EE than NPs prepared by the emulsion-evaporation method. In addition, the nanoprecipitation method is favored because it is a low-energy method with an easy operation that can be easily adapted to large-scale production batches [24]. The size and polydispersity (PDI) of BRP-187-loaded NPs as well as unloaded control NPs were analyzed after purification and after lyophilization, and the zeta potential (ζ) was measured after lyophilization (Table 1). The average hydrodynamic diameter (d_H_) of the final NPs was between 130 to 211 nm with PDI values of 0.09 to 0.28. PLGA NPs were in average up to 50 nm smaller than Acdex NPs.

**Table 1:** Overview of the NP properties

NP formulation	After purification		After lyophilization			EE (%)	LC (%)	PVA (%)
	d_H_ (nm)	PDI	d_H_ (nm)	PDI	ζ (mV)			
Acdex	210 ± 26	0.17 ± 0.07	211 ± 35	0.26 ± 0.09	− 12 ± 2	–	–	0.01 ± 0.000
Acdex[BRP-187]	196 ± 51	0.16 ± 0.11	178 ± 26	0.13 ± 0.07	− 13 ± 8	59 ± 23	1.7 ± 0.6	0.01 ± 0.000
Acdex-RhodB[BRP-187]	163 ± 15	0.20 ± 0.06	189 ± 37	0.28 ± 0.11	− 24 ± 2	67 ± 10	2.0 ± 0.3	n.m.
PLGA	124 ± 6	0.06 ± 0.03	130 ± 2	0.09 ± 0.03	− 20 ± 2	–	–	0.02 ± 0.002
PLGA[BRP-187]	153 ± 41	0.17 ± 0.14	158 ± 35	0.12 ± 0.06	− 15 ± 3	76 ± 22	2.2 ± 0.6	0.03 ± 0.002
PLGA-DY635	143 ± 3	0.08 ± 0.03	154 ± 5	0.15 ± 0.02	− 20 ± 1	–	–	0.02 ± 0.006
PLGA-DY635[BRP-187]	153 ± 2	0.12 ± 0.01	168 ± 9	0.19 ± 0.05	− 19 ± 1	87 ± 6	2.5 ± 0.2	0.02 ± 0.002

Concentration of NPs used for the PVA assay was 3 mg mL^−1^. SD for all measurements n ≥ 3. n.m.–not measured

*d*_*H*_ Hydrodynamic diameter obtained by DLS measurements. *EE* encapsulation efficiency. *LC* loading capacity

**Fig. 1:**
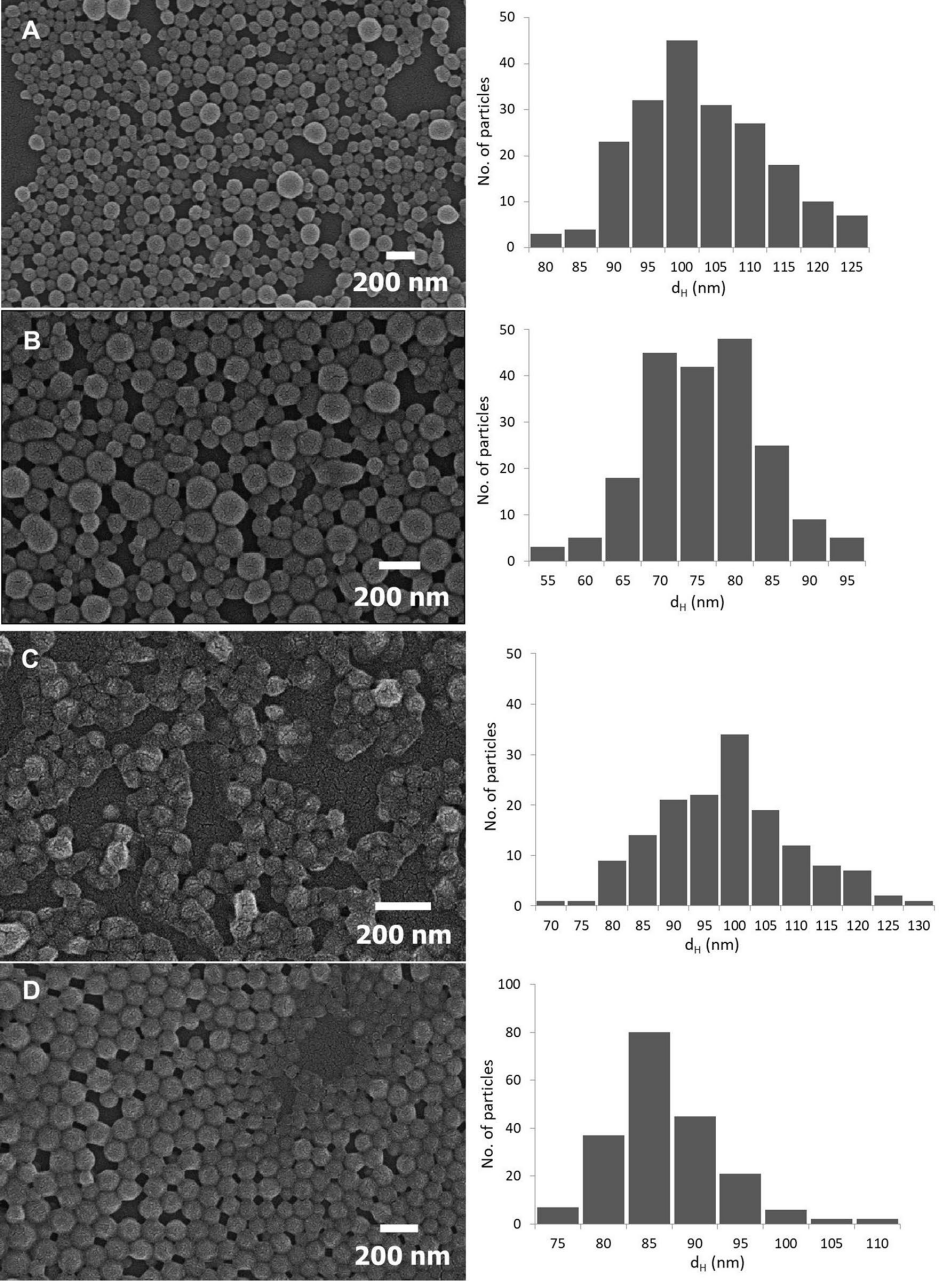
SEM images of NPs: Acdex (**a**), Acdex[BRP-187] (**b**), PLGA (**c**), PLGA[BRP-187] (**d**)

Scanning electron microscopy (SEM) imaging showed a spherical morphology of the NPs and smaller NP sizes compared to the results acquired by DLS, a common phenomenon when using orthogonal characterization techniques (Fig. 1) [25]. The size of the NPs measured by SEM was as follows: Acdex 95 ± 11 nm, Acdex[BRP-187] 73 ± 8 nm, PLGA 96 ± 11 nm, and PLGA[BRP-187] 84 ± 6 nm. Furthermore, EE of all NPs is given in Table 1 and was roughly 60% for Acdex particles and 80% for PLGA particles. Based on previous experiments, a drug-to-polymer content > 3% (w/w) fed in the formulation resulted in problems with the stability of the suspension (data not shown), a phenomenon that was also reported by others [26–28]. Meanwhile, the conditions used in this protocol (3%, w/w) were effective to encapsulate more than 60% of the drug without compromising the stability of the NPs. In addition, BRP-187 is a highly potent drug (IC_50(FLAP_) = 8 nM and IC_50(mPGES-1_) = 200 nM) [7], and a loading capacity of 1.7 to 2.5% corresponded to 37 to 55 µM of BRP-187 in 1 mg mL^−1^ NP suspension. Here, it was observed that Acdex formed larger particles but encapsulated less drug compared to PLGA, which is probably due to different drug-polymer interactions [26].

### Degradation profile of the nanoparticles

**Fig. 2:**
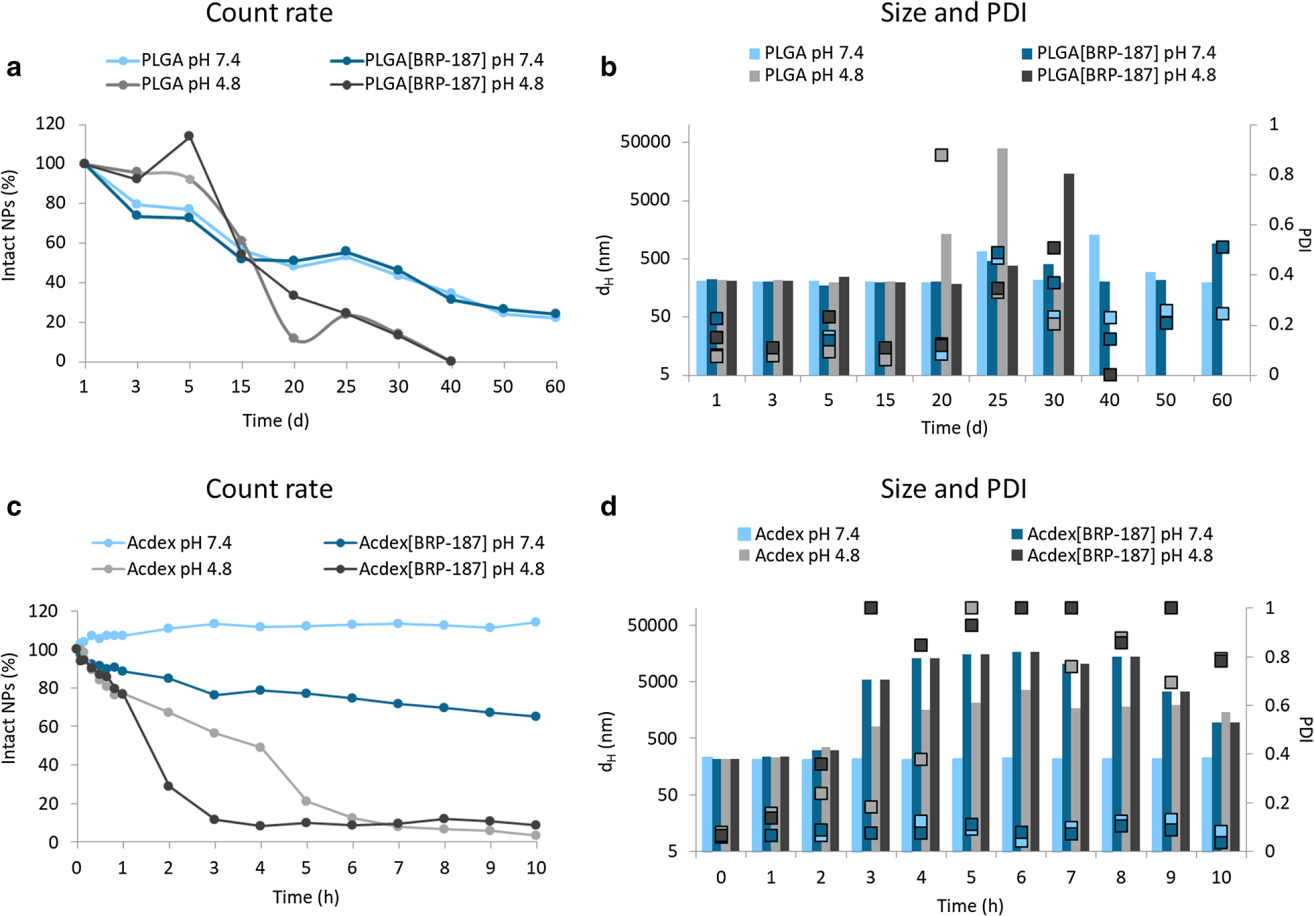
Degradation of NPs at physiological (7.4) and acidic (4.8) pH values: Acdex and Acdex[BRP-187] (**a**, **b**); and PLGA and PLGA[BRP-187] (**c**, **d**) measured by DLS (n = 1)

In DLS, the count rate corresponds to the number of the light photons detected in kilo-count per seconds (kcps), which is a good indicator of the quality of the measured sample [29]. A decreasing count rate indicates that less photons are detected *(*i.e. less light is scattered) [29] and, thus, less particles are present in a sample. In such a measurement, the NPs should show stable size and PDI values (100% intact NPs) at time point 0 of NPs incubated with buffer (Fig. 2a, c). As the NPs start to degrade, they steadily increase in size and polydispersity, which is a result of the degraded products dissolved in water (Fig. 2b, d). However, there is a chance that the aggregation of the NPs during the measurement might potentially take place based on the fact that the degradation products (dextran, acetone and methanol) might influence the conditions within the cuvette. This, in turn, might disturb the still intact NPs leading to a transient aggregation before they are completely degraded and solubilized in water. Aggregation that is only caused by ionic strength of the buffer solution can be neglected since experiments with the same buffer concentration but higher pH values do not show aggregation (Fig. 2d). Consequently, the count rate decreases over time as the degradation of the NPs proceeds.

The release of a molecule from a NP polymer matrix depends on several factors, i.e. the structure-property relationship between the drug and the polymer, hydrophobicity of the drug and the polymer, as well as the degradation rate, melting point and crystallinity of the polymer [30]. The degradation rate of the polymeric NPs in a cell-free environment was studied since it directly influences the release kinetics of the drug from the NP core. According to the literature, Acdex is an acid-labile polymer with a considerably higher pH sensitivity compared to the PLGA polymer [17, 31]. Our results revealed that after 10 h of incubation Acdex NPs exhibit good stability at pH 7.4 showing only swelling of the NPs, whereas at the same pH value the Acdex[BRP-187] NPs degraded to a degree of about 25%. Furthermore, at pH 4.8 after 2 h, the Acdex NPs degraded by only about 30%, whereas the Acdex[BRP-187] NPs degraded by 75% (Fig. 2c). This degradation behavior is suitable since NPs maintain 75% stability at physiological conditions. However, once internalized by the cells, the acidic environment of the endolysosome would trigger the degradation of Acdex, thereby releasing the drug. In case of PLGA, a complete degradation of NPs was observed at pH 4.8 within 40 days, whereas at pH 7.4, an 80% degradation was observed after 60 days (Fig. 2a), which is in line with previous studies [32–34]. Considering the degradation profile of the polymers in an acidic medium, the release of BRP-187 from Acdex NPs is expected to be fast due to the rapid degradation of the polymer, whereas the drug release from PLGA NPs is expected to be slower due to the diffusion of drug from the polymer matrix, since degradation of this polymer is very slow [35]. The degradation studies of the NPs at different pH values were investigated to obtain a first impression on the release of the drug from the NPs. However, it should be noted that such degradation profiles differ from the more complex environment of the endolysosomes [36].

### Residual amount of PVA

Previously, we described effects of surfactants on the stability of drug-loaded NPs, where we demonstrated that concentrations of ≤ 1% (w/v) PVA are desirable to formulate stable particles [10]. Here, we formulated NPs using 0.3% (w/v) PVA to obtain both suspension- and cryo-stability. The amount of residual PVA in the final NPs is listed in Table 1. The enzymatic- and pH-dependent degradation of the NPs relies not only on the properties of the polymer itself but is also strongly influenced by the digestibility of the surfactant. PVA coating can protect from enzymatic hydrolysis of the NPs by decreasing the wettability of the NPs [37], thus influencing the rate of degradation and drug release.

### Fluorescence dye-labeled nanoparticle uptake in PMNL

**Fig. 3:**
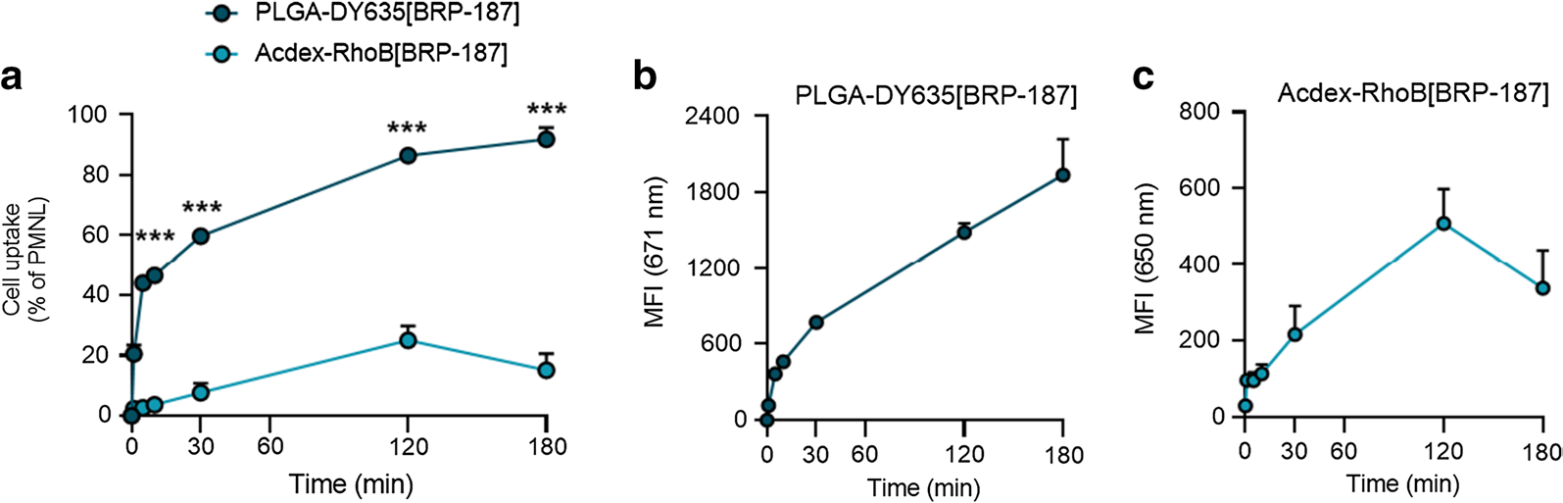
PMNL (1 × 10^6^) were incubated with 0.5 mg mL^−1^ PLGA-DY635[BRP-187] or Acdex-RhoB[BRP-187] NPs for the indicated time points at 37 °C. **a** Percentage of PMNL gated as positive due to the fluorescence signal of the dye-labeled NPs in the cell. **b**, **c** Mean fluorescence intensity (MFI) of NPs in the cell, measured by flow cytometry. For statistics analysis, a multiple t-test was used; p < 0.05 (*); p < 0.01 (**); p < 0.001 (***); n = 4

**Fig. 4:**
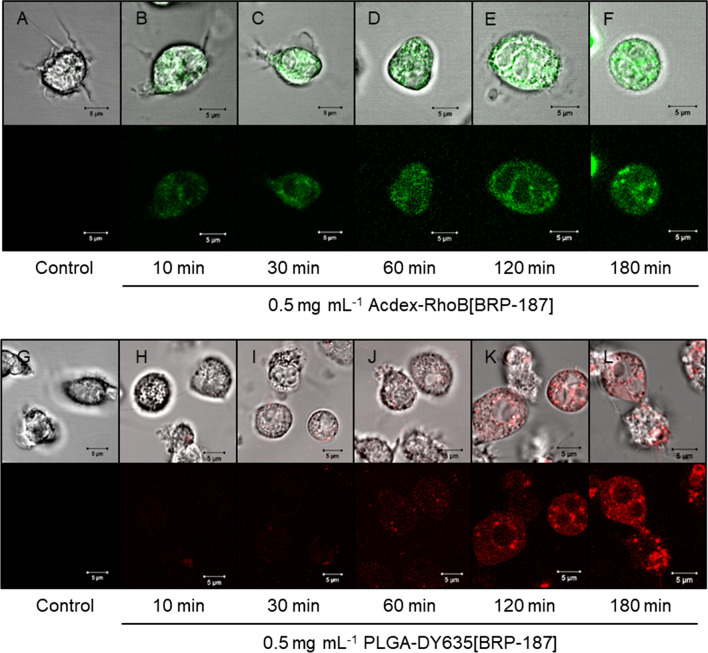
Confocal laser scanning microscopy of PMNL after isolation (**a**, **g**) and incubation at 37 °C with NPs for the indicated timepoints (**b**–**f** and **h**–**l**). Micrographs in the upper rows display the individual fluorescence channel while images in the bottom rows display the additional overlay of the transmitted light channel

PMNL (polymorphonuclear leukocytes) are pro-inflammatory innate immune cells that are abundant in the blood and produce substantial amounts of LTs and also PGE_2_ as targets for BRP-187 [7, 38]. Therefore, we used human PMNL as relevant cells to study the uptake of BRP-187-loaded NPs that were covalently labeled with fluorescent dyes (i.e., PLGA-DY635, Acdex-RhodB) for visualization in the cells. The dye-labeled NPs (loaded with BRP–187) were efficiently taken up by PMNL, which depends however on the nature of the polymer, being superior for PLGA over Acdex (Fig. 3). Within 10 min, 40% of the PMNL digested PLGA-DY635[BRP-187] NPs. After 180 min, approx. 85% of the PMNL took up these NPs along with a concomitant increase of the mean fluorescence intensity (MFI) per PMNL up to 1937 ± 283. Thus, PLGA NPs display an excellent cellular uptake. In contrast, Acdex-RhoB[BRP-187] NPs were taken up by only 23% of the PMNL after 120 min, with an even slightly lower uptake after 180 min, correlating with the MFI per cell over the entire time course (Fig. 3). According to the degradation profile of Acdex NPs (Fig. 2), the lower abundance might be a consequence of the concomitant degradation and elimination of the polymer inside the cell. After 2 h, approx. 70% of Acdex[BRP-187] NPs are degraded at the endolysosomal pH of 4.8. Therefore, the fluorescence signal may not increase further, even though NPs are still taken up, because the elimination of the labeled monomers from the cell is ongoing. However, both PLGA- and Acdex-based NPs are rapidly taken up by PMNL, even though PLGA NPs are ingested by a higher fraction of cells as compared to Acdex NPs, which might be also a consequence of the degradation. Additionally, we used confocal laser scanning microscopy to confirm the cellular internalization of NPs into PMNL (Fig. 4). PLGA-DY635[BRP-187] NPs show a time-dependent accumulation within the cells which appears as an increase of the intracellular fluorescence signal over time that is most prominent after 3 h. For Acdex-RhoB[BRP-187] NPs a comparable signal was already observed after 30 min. Remarkably, the NP-uptake after 30 min, as measured by the corresponding fluorescence intensity signal, does not further increase upon longer incubation up to 3 h.

### Encapsulation of BRP-187 into PLGA or Acdex NPs is not detrimental for target cells

**Fig. 5:**
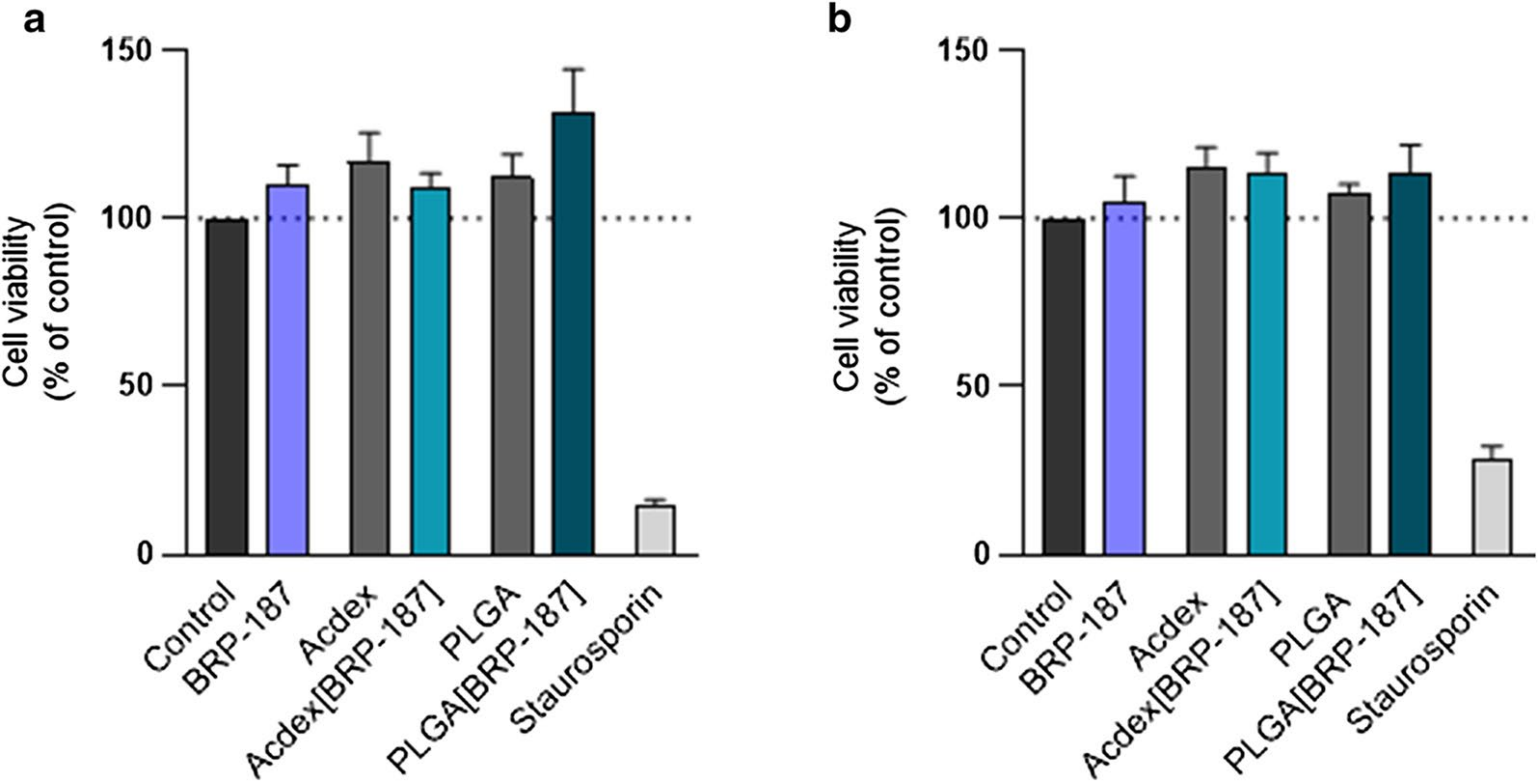
M1 macrophages (**a**) or M2 macrophages (**b**) were incubated with control (0.1% DMSO), free BRP-187 (10 µM), NP without drug, BRP-187 (10 µM) encapsulated into NP, or positive control (staurosporin 3 µM) for 24 h. Then, the release of LDH was analyzed using the Promega Cytotox 96^®^ assay. Values are normalized to the DMSO control (100%) and given as percentage of cell viability; data are means + S.E.M., n = 3

Next, we evaluated whether or not the NPs may cause detrimental effects upon long-term incubation (≥ 24 h) towards relevant target cells. Since PMNL are short-lived cells upon isolation being not suitable for long-term cytotoxicity tests, human monocyte-derived macrophages were used since they also possess the capability to produce PGE_2_ and LT [39]. None of the NP formulations (nor free BRP-187) showed detrimental effects on the viability of macrophages (with M1 or M2 phenotype) in terms of damage of the cell membrane over 24 h as measured by the lactate dehydrogenase (LDH) assay (Fig. 5). In supportive experiments using a RAW264.7 macrophage cell line that was incubated for 72 h with the NPs, an MTT assay revealed also no significant cytotoxic effects (data not shown). Thus, the NPs exert no detrimental effects against relevant target cells at concentrations used in functional assays.

### Effect of encapsulated BRP-187 on 5-LO product formation in PMNL

**Fig. 6:**
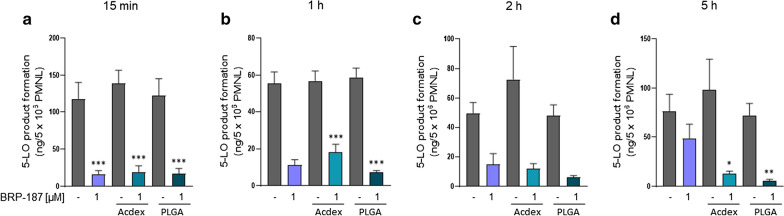
PMNL (5 × 10^6^) were preincubated with control (0.1% DMSO), BRP-187, Acdex, PLGA, Acdex[BRP-187] and PLGA[BRP-187] NPs for the indicated time points at 37 °C. The cells were then stimulated with 2.5 µM A23187 for 10 min, and 5-LO product formation was analyzed. Values are given as ng of 5-LO products (sum of the *trans*-isomers of leukotriene B_4_ (LTB_4_), LTB_4_ and 5-hydroxyeicosatetraenoic acid (5-HETE)). For statistical analysis, one-way ANOVA and Tukey’s multi comparison test was performed. p < 0.05 (*); p < 0.01 (**); p < 0.001 (***); n = 4

BRP-187 (1 µM) efficiently suppressed 5-LO product formation in isolated PMNL upon short preincubation periods ≤ 2 h (Fig. 6a–c), which is in agreement with our previous data [7]. However, after prolonged preincubation (5 h) with PMNL, the efficiency of BRP-187 (1 µM) was clearly reduced and suppression of 5-LO product formation was only 37 ± 5% of the control (Fig. 6d). Of interest, BRP-187 encapsulated into PLGA or Acdex NPs (corresponding to 1 µM BRP-187 as well) potently and consistently inhibited 5-LO product formation in PMNL by 80 to 92%, even after a preincubation period of 5 h. Note that NPs devoid of BRP-187 did not suppress 5-LO activity in PMNL. Therefore, we conclude that encapsulation of BRP-187 into PLGA or Acdex NPs generally accomplishes efficient inhibition of 5-LO product formation in PMNL, and, moreover, allows to overcome the loss of potency of BRP-187 upon prolonged exposure (i.e. 5 h) of PMNL.

### Effect of encapsulated BRP-187 on PGE_2_ formation in human M1 macrophages

**Fig. 7:**
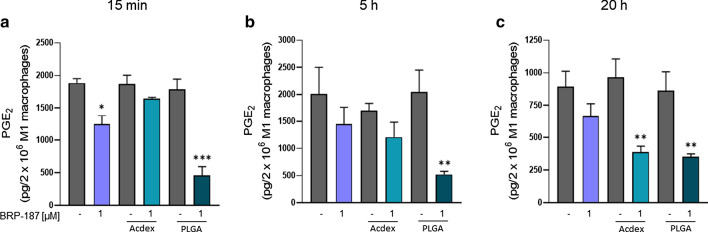
Determination of PGE_2_ formation in human macrophages: M1 macrophages (2 × 10^6^) were preincubated with BRP-187 or with BRP-187 encapsulated into Acdex or PLGA NPs for the indicated timepoints at 37 °C. Cells were then exposed to *E. coli* (O6:K2:H1), MOI = 50. After 90 min at 37 °C the reaction was stopped and PGE_2_ was analyzed after solid phase extraction (SPE) by UPLC-MS/MS. Values are given as pg of PGE_2_ per 2 × 10^6^ M1. For statistical analysis one-way ANOVA (p < 0.0001) and a Tukey‘s multi comparison test was performed. p < 0.05 (*); p < 0.01 (**); p < 0.001 (***); n = 3–4

Human M1 macrophages express high levels of mPGES-1 [40] and, upon exposure to pathogenic *E. coli*, produce high amounts of pro-inflammatory PGE_2_ [39]. Thus, *E. coli*-stimulated M1 macrophages are a suitable cell model to study the efficiency of mPGES-1 inhibitors. Note that many mPGES-1 inhibitors are highly potent in cell-free assays but markedly loose efficiency in cellular assays or in vivo [41], which necessitates technological approaches to overcome these hurdles. BRP-187 potently inhibited mPGES-1 in a cell-free assay (IC_50_ = 0.2 µM) [7], however, pretreatment of human M1 with 1 µM BRP-187 caused only moderate inhibition (30–37%) of *E. coli*-induced PGE_2_ formation regardless of the preincubation period (15 min, 5 or 20 h). In our study, encapsulation of BRP-187 into PLGA NPs strongly suppressed PGE_2_ levels at short (15 min) and prolonged (20 h) preincubation periods (Fig. 7). Also, Acdex[BRP-187] NPs caused strong reduction of PGE_2_ formation when M1 were preincubated for 20 h, while 15 min pretreatment was not effective. These data are in line with the cellular uptake pattern of the NPs, where PLGA NPs surpassed the uptake efficiency of Acdex NPs. Note that again, as for 5-LO product formation in PMNL, the empty NPs did not suppress PGE_2_ biosynthesis.

In summary, encapsulation of BRP-187 in PLGA and Acdex NPs overcomes the loss of effectiveness against mPGES-1 in intact cells versus cell-free assay conditions and confers the drug marked potency, highlighting this technological approach for efficient interference with pro-inflammatory PGE_2_ and LT formation in human cells. The beneficial effect of encapsulation of BRP-187 especially after prolonged incubations up to 20 h might be related to better stability and delayed release inside the cell. Intriguingly, encapsulation of BRP-187, particularly in PLGA-based NPs, accomplished efficient mPGES-1 inhibition in intact M1 macrophages, which was not the case for the free drug. It is conceivable that PLGA is cleaved in close proximity to the endoplasmic reticulum where mPGES-1 is located, thus, enabling unhindered access of BRP-187 to its target protein without being bound to other cellular membranes or cell compartments.

## Conclusion

Encapsulation of BRP-187 into polymer-based NPs improves the potency and duration of bioactivity of the drug in relevant human primary leukocytes compared to the free drug. PLGA and Acdex were chosen as biocompatible matrix polymers. Both polymers enabled stable formulations of BRP-187-loaded NPs with a monodisperse size distribution in the range of 200 nm and high EE according to a highly reproducible encapsulation method. It was shown that PLGA and Acdex NPs remained stable at physiological blood pH, whereas at pH 4.8, Acdex particles degraded very fast after 1 h, which indicates that they are biodegradable in the cellular endolysosome after they have been taken up via phagocytosis by PMNL or macrophages. According to the cellular uptake data, both kind of NPs are internalized by PMNL and started to degrade, leading to the release of BRP-187 inside the cell, though the uptake of PLGA NPs is faster and more efficient than Acdex NPs. Most importantly, both PLGA- and Acdex-based NPs loaded with BRP-187 are more efficient in suppressing 5-LO product formation and PGE_2_ biosynthesis in intact cells as compared to the free compound, particularly after prolonged preincubation periods. When isolated leukocytes were preincubated with BRP-187 for typical short-term periods, the compound was highly bioactive against FLAP [7], but prolonged exposure for more than 2 h markedly decreased the potency of BRP-187. Notably, encapsulation of BRP-187 in Acdex and PLGA particles accomplishes efficient mPGES-1 inhibition in M1 macrophages, which is a major step forward in the development of mPGES-1 inhibitors in general, since many mPGES-1 inhibitors fail in intact cells.

In view of the potential use of BRP-187 as drug for therapeutic treatment of chronic inflammatory diseases, the prolongation of its bioactivity is of utmost importance. An efficient encapsulation and release of BRP-187 is a promising approach to reach this aim. As a perspective, other biodegradable polymers for encapsulation of BRP-187 might be evaluated, and in addition to the properties reported here, further aspects of the nanoformulations (e.g. hydrophobicity, crystallinity and protein corona) might be assessed in more detail. It will also be challenging to study the effects of encapsulated BRP-187 in animal models of inflammation related to PGE_2_ and LTs in the future. In such physiological environment, the various aspects of bioavailability including distribution in other tissues and influence of plasma proteins may be assessed.

## Methods

### Materials

Poly(*D,L*-lactic-*co*-glycolic) acid (Resomer RG 502 H, copolymer composition of 50:50, 7–17 kDa, acid terminated) was purchased from Evonik Industries (Germany). Partially hydrolyzed PVA (Mowiol 4–88), acetone (> 99%) and dimethyl sulfoxide (DMSO > 99%, spectroscopic grade) were all purchased from Sigma-Aldrich (Germany). The dye DY635 was purchased from Dyomics (Jena, Germany). The covalent coupling of PLGA polymer with the dye DY635 amine was performed according to a standard procedure with 1-ethyl-3-(3-dimethylaminopropyl)carbodiimide (EDC) and *N*-hydroxysuccinimide (NHS) and was provided by SmartDyeLivery (Jena, Germany). The acetalation of dextran was done according to an adapted procedure (Mw of parent dextran 60 kDa), degree of substitution (DS) 2.97 (DS_cyclic acetal_ = 1.98, DS_acyclic acetal_ = 0.99) [19]. BRP-187 was synthesized according to an established protocol [42]. Deuterated and non-deuterated lipid mediator standards for UPLC-MS–MS quantification were purchased from Cayman Chemical/Biomol (Germany). For further materials, see specific experimental section.

### Acdex-rhodamine B synthesis

Rhodamine B was coupled to Acdex (Mw 9 to 11 kDa, Sigma Aldrich) according to an adapted procedure [43]: 1 g Acdex (6.17 mmol anhydroglucose unit, DS_acyclic acteal_ = 0.56, DS_cyclic acetal_ = 2.14) and 13 mg rhodamine B isothiocyanate were dissolved in 15 mL anhydrous pyridine and heated to 80 °C for 72 h under argon. The reaction mixture was precipitated in 150 mL distilled water, and centrifuged; the pellet was lyophilized. The resulting pink-colored powder was purified via gel permeation chromatography using BioBeads S-X1 in tetrahydrofuran (THF) to remove the free dye (yield 57%). Size-exclusion chromatography was performed in dimethylacetamide (DMAc) and 0.21% lithium chloride with a UV–VIS detector measuring at λ = 562 nm to prove the conjugation of the dye to the polymer.

### Nanoparticle formulation

Particles were prepared by nanoprecipitation using a syringe pump (Aladdin AL1000-220, World Precision Instruments, Berlin, Germany) with a flow rate of 2 mL min^−1^. First, 25 mg polymer (Acdex or PLGA) were dissolved in 5 mL acetone. For the drug solution, a 10 mg mL^−1^ stock of BRP-187 in DMSO was prepared and sonicated in an ultrasound bath for 15 min at room temperature. Subsequently, 75 µL of the drug solution were mixed with the polymer solution. For the aqueous phase, 40 mL of 0.3% (w/v) PVA solution were prepared. Further, the polymer (or polymer-drug) solution was injected into the aqueous solution, while stirring at 800 rpm at room temperature. After nanoprecipitation, the samples were stirred for 24 h in a fume hood to evaporate the acetone. The particles were washed once, using a Rotina 380 R centrifuge (Hettich Lab Technology, Germany) at 12.851×*g* for 60 min at 20 °C. After removing the supernatant, NPs were redispersed in 2.5 mL pure water, vortexed 5 to 10 s and then sonicated in an ultrasound bath for 30 min. For the Acdex NPs, 100 μL of 0.01% triethylamine (TEA) of pH 9 were added to the suspension. The NPs were stored at 4 °C overnight to allow complete dispersion in water. The concentration of the particle dispersions was determined by freeze-drying up to six aliquots of 100 or 200 μL NPs dispersion. The mass of the NPs was accurately weighed (Radwag Waagen, MYA 11.4Y, Germany) and an average was calculated for the NPs concentration.

### Dynamic light scattering (DLS)

The size, polydispersity index and the zeta-potential of the particles was measured using a Zetasizer Nano ZS with a laser wavelength of λ = 633 nm (Malvern Instruments, Germany). The measurements were performed at 173˚ backscatter angle with the following settings: Five repeated measurements at 25 °C, each measurement with five runs of 30 s and a 30 s equilibration time. The zeta-potential of the lyophilized NPs was measured at 25 °C with three repeated measurements. The NPs were characterized after purification (10 µL NP dispersion diluted in 1 mL pure water), and after lyophilization (100 µL of NP dispersion were lyophilized and redispersed in 1 mL pure water). The intensity size distribution is reported as the hydrodynamic diameter (d_H_) of the NPs.

The degradation behavior of Acdex and PLGA NPs was determined by monitoring the mean count rate (kcps). The NP samples were measured using fixed settings (37 °C, measurement position 4.65, and attenuator 7). The Acdex NPs were measured for 15 h: 180 measurements (delay between measurements 240 s), where each measurement consisted of three runs with 20 s run durations. The PLGA NPs were measured every day over a period of 60 days: Five measurements (with no delay between measurements), where each measurement consisted of one run with 30 s run duration.

### UV–VIS spectroscopy

For the calculating the encapsulation efficiency (EE) and loading capacity (LC) of the drug in the NPs, aliquots of 200 µL of the washed NP dispersion were lyophilized. The dry NP powder was dissolved in 200 µL DMSO (spectroscopic grade). The polymer-drug solution was measured at λ = 316 nm with 3 × 3 multiple reads per well and 2000 µm well border using the Infinite M200 Pro platereader (Tecan Group, Switzerland). For all EE measurements, a Hellma Quartz flat-transparent plate with 96 wells was used. A calibration curve of BRP-187 was obtained for each batch in the concentration range of 0.24 to µg mL^−1^ with R^2^ = 0.9997. Equations 1 and 2 were used to calculate EE and LC

1 $$\varvec{LC} = \frac{{\varvec{mass}\, \varvec{of} \varvec{drug} \,\varvec{recovered}}}{{\varvec{mass}\, \varvec{of} \varvec{particle} \,\varvec{recovered}}} \times 100$$

2 $$\varvec{EE} = \frac{{\varvec{LC}}}{{\varvec{LC \,theoretical}}} \times 100$$

### PVA assay

Determination of PVA in the NPs (%, w/w) was performed using UV–VIS spectroscopy. PVA forms a complex with iodine, which absorbs light at λ = 650 to 690 nm. In an adapted protocol, Lugol solution was used as iodine source [44]. Lyophilized NPs were redispersed in pure water (3 mg mL^−1^) and 90 µL were pipetted into a 96-well plate. Then, 20 μL of 1 M sodium hydroxide was added to each NP-containing well, and the solutions were mixed for 15 min at 850 rpm at room temperature. Next, 20 μL 1 M hydrochloric acid, 60 μL 0.65 M boric acid and 10 μL of Lugol solution were added to each well. Measurements on the plate reader were done at λ = 650 nm 15 min after the addition of the Lugol solution. The experiments were repeated three times for each NP formulation.

### Scanning electron microscopy (SEM)

Electron microscopy imaging was performed with a Sigma VP Field Emission Scanning Electron Microscope (Carl-Zeiss, Jena, Germany) using an InLens detector with an accelerating voltage of 6 kV. The samples were coated with a thin layer of platinum (4 nm) via sputter coating (CCU-010 HV, Safematic, Switzerland) before the measurement. ImageJ was used to measure the particle sizes from the images acquired by the SEM, where a mean diameter was deduced by measuring 150 to 200 NPs/image.

### Degradation study

The degradation behavior of loaded and unloaded NPs was tested at 37 °C in 0.05 M Tris–HCl buffer of pH 7.4 and 0.05 M acetate buffer of pH 4.8. Lyophilized NPs were dispersed in pure water (concentration of 3 to 7 mg mL^−1^). Next, NPs were mixed with buffer solution incubated at 37 °C and analyzed by DLS. Acdex NPs were measured for 15 h, whereas PLGA NPs were measured over 60 days (see Sect. “Fluorescence dye-labeled nanoparticle uptake in PMNL”). The degradation of the NPs was analyzed by monitoring the change in the mean count rate, size and PDI in DLS.

### Cell isolation and cell culture

Leukocyte concentrates were prepared from peripheral blood obtained from healthy human adult donors that received no anti-inflammatory treatment for the last 10 days (Institute of Transfusion Medicine, University Hospital Jena, Germany). The approval for the protocol was given by the ethical committee of the University Hospital Jena and all methods were performed in accordance with the relevant guidelines and regulations. To isolate PMNL and monocytes, the leukocyte concentrates were mixed with dextran (dextran from *Leuconostoc spp*. M_W_ ~ 40,000 g mol^−1^, Sigma Aldrich, Taufkirchen, Germany) for sedimentation of erythrocytes; the supernatant was centrifuged on lymphocyte separation medium (Histopaque^®^-1077, Sigma Aldrich). Contaminating erythrocytes in the pelleted PMNL were removed by hypotonic lysis using water. The pelleted PMNL were subsequently washed twice in ice-cold phosphate-buffered saline pH 7.4 (PBS) and finally resuspended in PBS. The peripheral blood mononuclear cell (PBMC) fraction on top of lymphocyte separation medium was washed with ice-cold PBS and seeded in cell culture flasks (Greiner Bio-one, Nuertingen, Germany) for 1.5 h (37 °C, 5% CO_2_) in PBS with Ca^2+^/Mg^2+^ (0133 g L^−1^/0,1 g L^−1^) to isolate monocytes by adherence. For differentiation and polarization of monocytes to M1 and M2 macrophages, we followed published procedures [39]. To obtain M1 macrophages, adherent monocytes were treated with 20 ng mL^−1^ granulocyte macrophage-colony stimulating factor (GM-CSF) Peprotech, Hamburg, Germany) for six days in RPMI 1640 supplemented with 10% fetal calf serum (FCS), 2 mmol L^−1^ l-glutamine, penicillin (100 U mL^−1^) and streptomycin (100 µg mL^−1^) for differentiation and were further incubated with 100 ng mL^−1^ lipopolysaccharide (LPS) and 20 ng mL^−1^ interferon-γ (Peprotech) for 48 h. M2 macrophages were obtained by treatment of monocytes with 20 ng mL^−1^ M-CSF (Peprotech) for 6 days, followed by 20 ng mL^−1^ IL-4 (Peprotech) for 48 h. Correct polarization and purity of macrophages was routinely checked by flow cytometry (FACS Canto Plus flow cytometer, BD Biosciences, Heidelberg, Germany) as reported [45] using the following antibodies: FITC anti-human CD14 (2 µg/test, clone M5E2, BD Biosciences), PE anti-human CD54 (1 µg/test, clone HA58, BD Biosciences), APC-H7 anti-human CD80 (0.25 µg/test, clone L307.4, BD Biosciences), PE-Cy7 anti-human CD163 (2 µg/test, clone RM3/1, Biolegend, San Diego, CA, USA), PerCP-eFluor710 anti-human CD206 (0.06 µg/test, clone 19.2, BD Biosciences, San Diego, CA, USA).

### Determination of 5-LO product formation in PMNL

For evaluation of the effects on 5-LO product formation in human PMNL, cells (5 × 10^6^ mL^−1^) were pre-incubated with BRP-187 or NPs (Acdex[blank], PLGA[blank], Acdex[BRP-187], PLGA[BRP-187]) for the indicated times (15 min up to 5 h) at 37 °C. Cells were then stimulated with 2.5 µM Ca^2+^-ionophore A23187 (Cayman, Ann Arbor, USA) for 10 min, and then the incubation was stopped with 1 mL ice-cold methanol containing 200 ng mL^−1^ PGB_1_ as internal standard. Samples were subjected to solid phase extraction and formed 5-LO products were separated and analyzed by RP-HPLC as described [46].

### Determination of prostaglandin E_2_ formation in human macrophages

Human monocyte-derived M1 macrophages (2 × 10^6^ cells) were seeded in 6-well-plates and preincubated for 15 min, 5 or 20 h with BRP-187 or NPs (Acdex[blank], PLGA[blank], Acdex[BRP-187], PLGA[BRP-187]) at 37 °C. The macrophages were subsequently incubated with pathogenic *E. coli* [O6:K2:H1] for 90 min. The reaction was stopped with ice-cold methanol containing deuterium-labeled internal standards (d8-5*S*-HETE, d4-LTB_4_, d5-LXA_4_, d5-RvD2, and d4-PGE_2_; 500 pg each). Samples were kept at − 20 °C for one day to allow protein precipitation. After centrifugation (2000×*g*, 4 °C, 10 min), 8 mL acidified water was added (final pH = 3.5) and samples were subjected to solid phase extraction using RP-18 columns and PGE_2_ was analyzed by UPLC-MS-MS exactly as described before  [45].

### Lactate dehydrogenase assay

The release of LDH from cells was analyzed using the CytoTox 96^®^ Non-Radioactive Cytotoxicity Assay (Promega GmbH, Mannheim, Germany). Briefly, 1 × 10^6^ M1 or M2 macrophages per well, suspended in RPMI-medium containing 10% FCS, penicillin/streptomycin and *l*-glutamine, were seeded in a 24-well plate. Lysis control and 0.2% triton X-100 were added to the cells and incubated for 45 min; compounds and control (0.1% DMSO) were added and incubated for 24 h at 37 °C. Stop solution was added, the plate was centrifuged (250×*g*, 4 min, room temperature) and 50 μL of supernatant from each well was transferred in a 96-well plate. Afterwards, 50 μL of substrate mixture was added and incubated for 30 min at room temperature in the dark. To finally stop the reaction, 50 μL of stop solution were added. The photometric measurement was performed at 490 nm using a Multiskan Spectrum plate reader (Thermo Fischer).

### Fluorescence dye-labeled nanoparticle uptake in PMNL

Time-dependent uptake of fluorescence dye-labeled NPs by PMNL was analyzed by flow cytometry and confocal laser scanning microscopy. Adherent PMNL (2 × 10^6^) were incubated for the indicated time points with 0.5 mg mL^−1^ labeled NPs (PLGA-DY635[BRP-187] or Acdex-RhoB[BRP-187]). For flow cytometry, cells were washed once with PBS containing 0.5% BSA and incubated with PBA-E (PBS with 0.5% BSA, 2 mM EDTA and 0.1% sodium azide) containing 0.4% lidocaine for detaching. PMNL containing fluorescently stained NPs were analyzed by flow cytometry using BD LSR Fortessa (BD Bioscience). The red laser (644 nm) in combination with 670|14 filters for DY635 labeled NPs and the violet laser (405 nm) in combination with 655|8 filters for rhodamine B labeled NPs were used for flow cytometric analysis. Data were analyzed using FlowJo X Software (BD Bioscience).

For confocal laser scanning microscopy, PMNL were (i) washed once with PBS after incubation with the respective NPs for the indicated time points as described above for flow cytometry, (ii) submerged with phenol red-free RPMI 1640 and (iii) subsequently subjected to microscopic analysis. CLSM images were acquired using a Zeiss LSM 880 (Carl Zeiss, Oberkochen, Germany) with following settings: PLGA NPs labeled with DY635: λ_Ex_ = 633 nm, λ_Em_ = 638 to 759 nm and transmission signal with PMT detector; Acdex NPs labeled with rhodamine B: λ_Ex_ = 514 nm, λ_Em_ = 531 to 703 nm and transmission signal with PMT detector. Images were captured with an iLCI Plan-Neofluar 63 × objective using identical settings for image acquisition within experimental groups.

## Data Availability

Not applicable.

## Abbreviations

5-LO: 5-Lipoxygenase
5S-HETE: 5-Hydroxyeicosatetraenoic acid
AA: Arachidonic acid
Acdex: Acetalated dextran
Acdex-RhodB: Acetalated dextran coupled to Rhodamine B
BSA: Bovine serum albumin
CLSM: Confocal laser scanning microscopy
COX: Cyclooxygenase
d_H_: Hydrodynamic diameter
DLS: Dynamic light scattering
DMAc: Dimethylacetamide
DMF: Dimethylformamide
DMSO: Dimethylsulfoxide
EDC: 1-Ethyl-3-(3-dimethylaminopropyl)carbodiimide
EDTA: Ethylenediaminetetraacetic acid
EE: Encapsulation efficiency
FCS: Fetal calf serum
FLAP: 5-Lipoxygenase-activating protein
GM-CSF: Granulocyte macrophage-colony stimulating factor
IC_50_: Half maximal inhibitory concentration
ICH: International council for harmonization of technical requirements for pharmaceuticals for human use
IL-4: Interleukin 4
LC: Loading capacity
LDH: Lactate dehydrogenase
LT: Leukotrienes
LTB_4_: Leukotriene B_4_
LXA4: Lipoxin A_4_
MFI: Mean fluorescence intensity
mPGES-1: Microsomal prostaglandin E_2_ synthase-1
MTT: 3-(4,5-Dimethylthiazol-2-yl)-2,5-diphenyltetrazolium bromide
NHs: *N*-Hydroxysuccinimide
NPs: Nanoparticles
NSAIDs: Nonsteroidal anti-inflamamtory drugs
PBA-E: PBS with 0.5% BSA, 2 mM EDTA and 0.1% sodium azide
PBMC: Peripheral blood mononuclear cell
PBS: Phosphate buffer saline
PDI: Polydispersity index
PG: Prostaglandins
PGB_1_: Prostaglandin B_1_
PGE_2_: Prostaglandin E_2_
PGH_2_: Prostaglandin H_2_
PLGA: Poly(lactic-*co*-glycolic) acid
PMNL: Polymorphonuclear leukocytes
PVA: Poly (vinyl alcohol)
RP-HPLC: Reverse phase-high performance liquid chromatography
RvD2: Resolvin D2
SEM: Scanning electron microscope
SPE: Solid phase extraction
TEA: Triethylamine
THF: Tetrahydrofuran
UPLC-MS/MS: Ultra performance liquid chromatography tandem mass spectrometry
