# Correction: Encapsulation of the dual FLAP/mPEGS‑1 inhibitor BRP‑187 into acetalated dextran and PLGA nanoparticles improves its cellular bioactivity

**DOI:** 10.1186/s12951-024-02341-7

**Published:** 2024-02-27

**Authors:** Blerina Shkodra, Christian Kretzer, Paul M. Jordan, Paul Klemm, Andreas Koeberle, David Pretzel, Erden Banoglu, Stefan Lorkowski, Maria Wallert, Stephanie Höppener, Steffi Stumpf, Antje Vollrath, Stephanie Schubert, Oliver Werz, Ulrich S. Schubert

**Affiliations:** 1 Laboratory of Organic and Macromolecular Chemistry (IOMC), Friedrich Schiller University Jena, Humboldtstraße 10, 07743 Jena, Germany; 2 Jena Center for Soft Matter (JCSM), Friedrich Schiller University Jena, Philosophenweg 7, 07743 Jena, Germany; 3 Department of Pharmaceutical/Medicinal Chemistry, Institute of Pharmacy, Friedrich Schiller University Jena, Philosophenweg 14, 07743 Jena, Germany; 4 Department of Pharmaceutical Chemistry, Faculty of Pharmacy, Gazi University, Etiler, Yenimahalle, 06330 Ankara, Turkey; 5 Institute of Nutritional Sciences, Friedrich Schiller University Jena, Dornburger Straße 25, 07743 Jena, Germany; 6 Department of Pharmaceutical Technology and Biopharmacy, Institute of Pharmacy, Friedrich Schiller University Jena, Lessingstraße 8, 07743 Jena, Germany; 7 Michael Popp Reseach Institute, University of Innsbruck, Mitterweg 24, 6020 Innsbruck, Austria

**Correction: J Nanobiotechnol (2020) 18:73** 10.1186/s12951-020-00620-7

In this article the author name Blerina Shkodra was incorrectly written as Blerina Shkodra-Pula.

The original article [1] has been revised.
